# Distress and pleasure indicators in health care workers on the COVID-19 front line

**DOI:** 10.1590/1518-8345.5707.3519

**Published:** 2022-04-20

**Authors:** Patrícia Campos Pavan Baptista, Daniela Campos de Andrade Lourenção, João Silvestre, Arthur Arantes da Cunha, Cristiane Helena Gallasch

**Affiliations:** 1 Universidade de São Paulo, Escola de Enfermagem, São Paulo, SP, Brasil.; 2 Bolsista da Coordenação de Aperfeiçoamento de Pessoal de Nível Superior (CAPES), Brasil.; 3 Centro Universitário São Camilo, Departamento de Medicina, São Paulo, SP, Brasil.; 4 Universidade Federal do Amapá, Departamento de Ciências Biológicas e da Saúde, Macapá, AP, Brasil.; 5 Universidade do Estado do Rio de Janeiro, Faculdade de Enfermagem, Rio de Janeiro, RJ, Brasil

**Keywords:** Health Personnel, COVID-19, Psychological Distress, Plesure, COVID-19 Pandemic, Saúde do Trabalhador, COVID-19, Sofrimento Psicológico, Prazer, Pessoal de Saúde, Pandemia por COVID-19, Salud Laboral, COVID-19, Distrés Psicológico, Placer, Personal de Salud, Pandemia de COVID-19

## Abstract

**Objective:**

to evaluate distress and pleasure indicators in health care workers on the front line of care for suspected or confirmed COVID-19 cases.

**Method:**

an exploratory, analytical and cross-sectional study with a quantitative approach. The studied sample consisted of 437 health professionals invited by electronic means, who answered the questionnaire on sociodemographic information, occupational aspects and clinical conditions. Distress and pleasure at work were considered as outcomes, which were analyzed with multinomial logistic regression regarding the associated independent variables.

**Results:**

Most of the participants were female (71.0%), nurses (55.6%), with a weekly working shift of 40 hours or more (75.8%); 61.6% of the participants suffered from mental distress. The psychosocial characteristics of high-strain work and low social support were reported by 23.8% and 52.9% of the participants, respectively. In the multiple analysis, distress and lack of pleasure at work were associated with high job strain, low support from co-workers and mental distress. The profession is also associated with distress at work.

**Conclusion:**

distress and lack of pleasure at work are associated with occupational characteristics and mental strain among health care workers in the COVID-19 scenario.

## Resumo

**Objetivo:**

avaliar indicadores de sofrimento e prazer em trabalhadores de saúde na linha de frente do cuidado aos casos suspeitos ou confirmados de COVID-19.

**Método:**

estudo exploratório, analítico, transversal, com abordagem quantitativa. A amostra estudada foi constituída por 437 profissionais da saúde convidados por meio eletrônico, que responderam ao questionário sobre informações sociodemográfica, aspectos ocupacionais e condições clínicas. Foram considerados como desfechos o sofrimento e o prazer no trabalho, os quais foram analisados com regressão logística multinomial quanto às variáveis independentes associadas.

**Resultados:**

a maioria dos participantes era do sexo feminino (71,0%), enfermeira (55,6%), com jornada de trabalho semanal de 40 horas ou mais (75,8%); 61,6% dos participantes estavam em sofrimento mental. As características psicossociais do trabalho de alta exigência e de baixo apoio social foram informadas, respectivamente, por 23,8% e 52,9% dos participantes. Na análise múltipla, o sofrimento e a falta de prazer no trabalho estiveram associados com a alta exigência no trabalho, baixo apoio dos colegas de trabalho e sofrimento mental. A profissão também está associada ao sofrimento no trabalho.

**Conclusão:**

o sofrimento e a falta de prazer no trabalho estão associados às características ocupacionais e ao desgaste mental entre trabalhadores da saúde no cenário da COVID-19.

## Resumen

**Objetivo:**

evaluar indicadores de sufrimiento y placer en trabajadores de la salud en la primera línea de atención de casos sospechosos o confirmados de COVID-19.

**Método:**

estudio exploratorio, analítico, transversal con enfoque cuantitativo. La muestra estudiada estuvo conformada por 437 profesionales de la salud invitados por medios electrónicos, que respondieron el cuestionario sobre información sociodemográfica, aspectos ocupacionales y condiciones clínicas. Se consideraron como resultados el sufrimiento y el placer en el trabajo, que fueron analizados con regresión logística multinomial en cuanto a las variables independientes asociadas.

**Resultados:**

la mayoría de los participantes era de sexo femenino (71,0%), enfermera (55,6%), tenía una jornada laboral semanal de 40 horas o más (75,8%); el 61,6% de los participantes presentaban sufrimiento mental. En cuanto a las características psicosociales del trabajo, los participantes lo consideraron de alta exigencia y bajo apoyo social, 23,8% y 52,9% respectivamente. En el análisis múltiple, la angustia y la falta de placer en el trabajo se asociaron con una alta exigencia laboral, poco apoyo de los compañeros de trabajo y sufrimiento mental. La profesión también está asociada al sufrimiento en el trabajo.

**Conclusión:**

el sufrimiento y la falta de placer en el trabajo se asocian con características ocupacionales y el agotamiento mental en los trabajadores de la salud en el escenario del COVID-19.

**Highlights**

**(1)** Critical levels of distress at work and lack of recognition were evidenced.

**(2)** Being a nursing technician/assistant was associated with distress at work.

**(3)** High pleasure in professional fulfillment was evidenced.

**(4)** There was an association between high-strain work and low social support with low pleasure.

**(5)** Low pleasure potential in freedom of expression was identified.

## Introduction

The study of the relationship between man and work and its consequences for mental health highlights the role of a French thought current called Psychopathology of Work, which was built based on the conceptions and research developed by Christophe Dejours^1^, called “Psychodynamics of Work”. In this approach, studies involving health care workers stand out, due to their exposure to workloads and all the consequences arising from a professional routine that requires knowledge, technical skills and constant attention. In addition to that, it is characterized by daily contact with pain, suffering and losses, translated into experiences of pleasure and distress.

A number of research studies have been conducted to identify the impact of the pandemic on people’s lives around the world. Among those who have undergone changes in their work routines, health care workers, involved in the care of suspected or confirmed COVID-19 patients, were called the front line in the fight against the pandemic. 

In the Brazilian context, given the COVID-19 pandemic, there is a worrying scenario in the work routines of workers on the front line in the fight against the pandemic involved in the care of patients with suspected or confirmed infection. Changes led to the worsening of work precariousness, with shortage of personnel and of Personal Protective Equipment, as well as fragility in labor ties, in addition to a scenario in which emotions such as fear of dying, concern about contaminating family members and anxiety for not knowing what the following day would be like, tend to intensify the emotional pressure they experience^2^.

A recent study involving Nursing professionals showed that, in this pandemic context, stress, burnout, and moral distress can be accentuated, with negative repercussions on the workers’ physical and psychological health^3^ ^-^ ^4^. On the other hand, interpersonal relationships in health teams make it possible to transform the perception of the realities experienced and the professional identity itself, which can promote pleasure in these professionals^5^.

Work is a strengthening element of people’s health and develops the perception of pleasure, with important evidence that positive aspects should be valued in the management of human resources in health^6^. Among the health professionals, the complexity involved in caring for suspected or confirmed COVID-19 patients is responsible for an excessive demand of personal resources from the teams, turning this context into a risk to the workers’ health. 

It is in this worrying and challenging scenario, but also propitious to the development of knowledge about positive aspects of professional strengthening, that this research is inserted, with the objective of evaluating distress and pleasure indicators in health care workers on the front line of the care provided for suspected or confirmed COVID-19 cases. 

## Method

### Type of study

This is an exploratory, analytical and cross-sectional study with a quantitative approach. The STROBE (Strengthening the Reporting of Observational Studies in Epidemiology) script for observational studies was followed, as recommended by the EQUATOR network.

### Participants

Due to the limitations of face-to-face access to the potential participants and institutions, in the first half of 2020, due to the pandemic, the sample was assembled by convenience, considering any health professional in the Brazilian territory who was active in the front line of the care provided to suspected or confirmed COVID-19 patients, at any care level and in any part of the Brazilian territory. A total 437 workers answered the invitation.

### Data collection

The invitation took place by the snowball method, starting from the researchers’ contact database, with virtual dissemination via email and social networks between April and June 2020. In the invitation, the sole research selection criterion was included, which was professional practice in assisting suspected or confirmed COVID-19 cases. The website address contained a form with the following information: a) sociodemographic data (gender, age at the time of the research, federal unit of residence/work); b) occupational aspects (current profession, health care level in which the person works, number and nature of the institution(s) with which the person has an employment contract, type(s) of labor contract, weekly working hours, psychosocial characteristics of work, perception of distress and pleasure at work) and c) clinical issues (history of morbidities, mental distress). 

### Research instruments

As for the psychosocial characteristics of work, the version of the *Job Stress Scale* for Portuguese spoken in Brazil was used, which measures the participants’ perception of psychosocial factors at work, considering three dimensions: task demands, control over their fulfillment and support from supervisors and co-workers^7^. The questionnaire contains 17 items, with four answer options on a Likert scale, to assess the three dimensions^8^. The interaction between demands and control allows classifying the working conditions according to quadrants, as follows: active work (high demand and high control), passive work (low demand and low control), low-strain work (low demand and high control) and high-strain work (high demand and low control). This last quadrant is considered the most harmful for the worker. The cutoff points established to delimit them were the midpoint of each dimension: 12.5 for demand; 15.5 for control. The dichotomization of social support into high/low was also performed from the midpoint of the scale (15.5).

The Distress and Pleasure at Work Indicators Scale (*Escala de Indicadores de Prazer e Sofrimento no Trabalho*, EIPST) is one of the four *scales* that make up the Brazilian questionnaire called Inventory on *Work* and Risks of Illness (*Inventário sobre o Trabalho e Riscos de Adoecimento*, ITRA). EIPST consists of four factors: two to assess pleasure (freedom of expression and professional fulfillment) and two to assess distress (professional burnout and lack of recognition). The pleasure domain score allows classifying the condition as satisfactory (above 4.0), critical (between 3.9 and 2.1) or severe (equal to or less than 2.0). The distress domain score allows classifying the condition as severe (above 4.0), critical (between 3.9 and 2.1) and satisfactory (equal to or less than 2.0)^9^.

To assess mental distress, the Brazilian version of the *Self-Reporting Questionnaire*(SRQ-20) was used, with 20 questions about depression, anxiety and stress symptoms and dichotomous answers (yes/no). Mental distress is considered when the participant answers positively to seven or more questions^10^ ^-^ ^12^.

### Data analysis

The continuous variables were presented using descriptive statistics (frequencies, mean values and standard deviations), being categorized for analysis. Distress and pleasure at work were considered as outcomes. The categorical variables were submitted to the Chi-square or Fisher’s exact tests, with each outcome. Variables whose p-value was equal to or less than 0.20 were selected for the multinomial logistic regression model. The final model was built with inclusion of the variables according to the increasing order of the p-value. The stepwise forward method was used, keeping in the modeling the variables associated with at least one of the outcome categories (critical and severe) (p<0.05), always considering the satisfactory category as a reference for analysis. The data were tabulated in Microsoft Excel® for Office 365 MSO spreadsheets (version 16.0.12527.20986) and analyzed using the Statistical Package for the Social Sciences® statistical software, version 20.0.

### Ethical aspects

The research protocol followed the recommendations set forth in Resolution No. 466/2012 of the National Health Council, in addition to its complementary resolutions, being registered in *Plataforma Brasil* (CAAE 30599420.0.0000.0008) and approved by the National Research Ethics Commission (*Comissão Nacional de Ética em Pesquisa*, CONEP), under opinion No. 3,979,223/2020. All the participants virtually accessed the Free and Informed Consent Form (FICF) and accepted to take part in the research before having access to the questionnaire. After completion of participation, all received the informed consent signed by the responsible researcher by email.

## Results

The studied sample consisted of 437 health professionals. According to Table 1, the majority were female, nurses, with a weekly workday of 40 hours or more and without morbidities. The mean age was 38.4 years old (median of 37; standard deviation + 9.95). As for the psychosocial characteristics of work, high demand and low social support were reported by 23.8% and 52.9% of the participants, respectively. Most suffered from mental distress at the time of the research.

**Table 1:** Distribution of the health care workers according to sociodemographic, occupational and clinical characteristics. Brazil, 2020 (n=437)

Variable (n)	n (%)
Gender (434^*^)	
Male	126 (29.0)
Female	308 (71.0)
**Age group in years old (437)**	
20 - 29	90 (20.6)
30 - 39	161 (36.8)
40 - 49	124 (28.4)
50 - 59	49 (11.2)
60 or more	13 (3.0)
**Region (437)**	
North	79 (18.1)
Northeast	30 (6.9)
Midwest	8 (1.8)
Southeast	300 (68.6)
South	20 (4.6)
**Profession (437)**	
Nurses	243 (55.6)
Nursing Tech./Assist.^†^	41 (9.4)
Physicians	69 (15.8)
Physiotherapists	22 (5.0)
Psychologists	15 (3.4)
Others	47 (10.8)
Number of institutions where they work (435^*^)	
One	265 (60.9)
Two	129 (29.7)
Three	24 (5.5)
Four or more	17 (3.9)
Nature of the institution (432^*^)	
Only public	303 (70.1)
Only private	79 (18.3)
Mixed	50 (11.6)
Type of employment contract (435^*^)	
Only statutory	136 (31.3)
Only permanent	145 (33.3)
Only temporary	45 (10.3)
Statutory and celetary	16 (3.7)
Others	93 (21.4)
Health care level (428^*^)	
Primary	135 (31.5)
Secondary	79 (18.5)
Tertiary	129 (30.1)
Quaternary	20 (4.7)
More than one level	65 (15.2)
Weekly working hours (434^*^)	
< 20	17 (3.9)
20 - 39	88 (20.3)
40 - 59	214 (49.3)
60 or more	115 (26.5)
**Morbidity (437)**	
Yes	158 (36.2)
No	279 (63.8)
**Mental distress (437)**	
Yes	269 (61.6)
No	168 (38.4)
**Demand-control (437)**	
Active work	58 (13.3)
Passive work	128 (29.3)
High-strain	104 (23.8)
Low-strain	147 (33.6)
**Support at work (437)**	
High	206 (47.1)
Low	231 (52.9)

^*^Number of participants who answered this question from the questionnaire; ^†^Nursing Tech./Assist. = Nursing Technician or Assistant

As for distress at work, the mean score presented critical levels, with severe professional burnout and lack of critical recognition, as shown in Table 2. Table 3 presents the statistically significant variables in the analysis of the distribution of the independent variables and the distress categories selected for the multiple analysis: profession, demand-control, social support at work and mental distress.

**Table 2:** Descriptive statistics and risk classification according to indicators and factors of the Pleasure and Distress at Work Scale. Brazil, 2020 (n=437)

Indicators and factors (n)	Mean	Standard Deviation	Classification
**Pleasure**	3.9	±1.2	Critical
Professional fulfillment	4.1	±1.3	Satisfactory
Freedom of expression	3.8	±1.3	Critical
**Distress**	3.4	±1.4	Critical
Professional exhaustion	4.0	±1.3	Severe
Lack of recognition	2.9	±1.7	Critical

As for pleasure at work, Table 2 shows that the mean score indicated a critical level, with the professional fulfillment factor at a satisfactory level and the freedom of expression factor at a critical level. In the distribution analysis according to the categories of this dimension, Table 3 shows the statistically significant variables: demand-control, social support at work and mental distress, which were selected for multiple logistic modeling.

**Table 3:** Distribution of the health care workers according to sociodemographic, occupational and clinical characteristics as per the risk classifications of the Pleasure and Distress at Work Scale. Brazil, 2020 (n=437)

Variable (n)	Pleasure			p-value	Distress			p-value
	Satisfactory	Critical	Severe		Satisfactory	Critical	Severe	
Gender (434^*^)				0.390^†^				0.602^†^
Male	72	43	11		27	55	44	
Female	161	126	21		54	146	108	
**Age group in years old (437)**				0.054^†^				0.041^†^
20 - 29	48	38	4		19	42	29	
30 - 39	81	63	17		25	74	62	
40 - 49	63	56	5		19	56	49	
50 - 59	31	12	6		12	25	12	
60 or more	10	3	0		6	7	0	
**Region (437)**				0.397^‡^				0.197^‡^
North	46	30	3		15	40	24	
Northeast	19	9	2		7	14	9	
Midwest	2	5	1		0	4	4	
Southeast	155	119	26		59	132	109	
South	11	9	0		0	14	6	
**Profession (437)**				0.046^†^				0.005^†^
Nurses	129	97	17		41	118	84	
Nursing Tech./Assist.^‡^	16	20	5		2	19	20	
Physicians	44	21	4		19	32	18	
Physiotherapists	8	11	3		2	10	10	
Psychologists	13	1	1		8	4	3	
Others	23	22	2		9	21	17	
Number of institutions where they work (435^*^)				0.416^†^				0.799^†^
One	134	110	21		52	127	86	
Two	71	51	7		20	57	52	
Three	15	7	2		4	11	9	
Four or more	12	3	2		4	8	5	
Nature of the institution (432^*^)				0.739^†^				0.581^†^
Only public	163	120	20		53	145	105	
Only private	38	34	7		19	31	29	
Mixed	29	17	4		8	25	17	
Type of employment contract (435^*^)				0.690^†^				0.912^†^
Only statutory	71	56	9		22	65	49	
Only permanent	72	63	10		27	68	50	
Only temporary	25	15	5		9	20	16	
Statutory and celetary	9	7	0		2	6	8	
Others	56	31	6		20	45	28	
Health care level (428^*^)				0.042^†^				0.925^†^
Primary	71	55	9		23	67	45	
Secondary	42	34	3		15	37	27	
Tertiary	70	53	6		23	61	45	
Quaternary	10	5	5		5	6	9	
More than one level	36	21	8		14	29	22	
Weekly working in hours (434^*^)				0.548^†^				0.164^†^
< 20	9	6	2		6	7	4	
20 - 39	40	39	9		17	37	34	
40 - 59	116	82	16		41	109	64	
60 or more	66	44	5		17	50	48	
**Morbidity (437)**				0.711^†^				0.022^†^
Yes	80	66	12		24	66	68	
No	153	106	20		57	138	84	
**Mental distress (437)**				<0.001^†^				<0.001^†^
Yes	107	136	26		17	117	135	
No	126	36	6		64	87	17	
**Demand-control model (437)**				<0.001^†^				<0.001^†^
Active work	34	20	4		8	26	24	
Passive work	53	64	11		15	68	45	
High-strain	29	59	16		3	36	65	
Low-strain	117	29	1		55	74	18	
**Support at work (437)**				<0.001^†^				<0.001^†^
High	159	44	3		64	103	39	
Low	74	128	29		17	101	113	

^*^Number of participants who answered this question from the questionnaire; ^†^Chi-square test; ^‡^Fisher’s Exact test; ^§^Nursing Tech./Assist. = Nursing Technician or Assistant

Table 4 presents the final multinomial model of distress at work, which showed that nursing technicians and assistants were more likely to have severe conditions than other categories of health care workers. The perception of a high-strain and low social support job increased the chance of critical and severe distress levels, as well as the state of mental distress. 

As for pleasure at work, Table 5 shows that participants with high-strain work were more likely to have critical or severe conditions of scarce pleasure at work than other psychosocial conditions. In addition to that, the perception of low social support at work was associated with a greater chance of critical and severe conditions of reduced pleasure at work. On the other hand, mental distress was only associated with critical conditions.

**Table 4:** Multinomial, univariate and multiple logistic regression analysis to study the factors associated with distress at work in health care workers active in the COVID-19 context. Brazil, 2020 (n=437)

Variable (n)	Univariate						Multiple					
	Critical			Severe			Critical			Severe		
	OR^*^	95% CI^†^	p-value	OR^*^	95% CI^†^	p-value	OR^*^	95% CI^†^	p-value	OR^*^	95% CI^†^	p-value
**Gender**												
Male	0.75	0.43 - 1.31	0.319	0.81	0.46 - 1.46	0.489	0.87	0.47 - 1.62	0.665	1.15	0.55 - 2.41	0.711
Female	1	---	---	1	---	---	1	---	---	1	---	---
**Age group in years old**												
Up to 37	1.3	0.77 - 2.18	0.32	1.39	0.81 - 2.39	0.235	0.99	0.56 - 1.77	0.975	0.97	0.49 - 1.92	0.925
38 or more	1	---	---	1	---	---	1	---	---	1	---	---
**Profession**												
Nursing Tech./Assist.^‡^	4.06	0.92 - 17.83	0.064	5.98	1.36 - 26.30	0.018	3.78	0.80 - 17.85	0.093	7.15	1.39 - 36.67	0.018
Others	1	---	---	1	---	---	1	---	---	1	---	---
**Mental distress**												
Yes	5.06	2.77 - 9.25	<0.001	29.9	14.33 - 62.36	<0.001	4.03	2.14 - 7.61	<0.001	19.89	8.94 - 44.29	<0.001
No	1	---	---	1	---	---	1	---	---	1	---	---
**Demand-control**												
High-strain	5.57	1.66 - 18.65	0.005	19.42	5.87 - 64.31	<0.001	4.48	1.27 - 15.81	0.02	12.22	3.34 - 44.71	<0.001
Others	1	---	---	1	---	---	1	---	---	1	---	---
**Support at work**												
High	1	---	---	1	---	---	1	---	---	1	---	---
Low	3.69	2.02 - 6.73	<0.001	10.91	5.71 - 20.83	<0.001	2.84	1.51 - 5.35	0.001	6.36	3.05 - 13.24	<0.001

* OR = Odds ratio; ^†^95% CI = 95% Confidence Interval; ^‡^Nursing Tech./Assist. = Nursing Technician or Assistant

**Table 5:** Multinomial, univariate and multiple logistic regression analysis to study the factors associated with pleasure at work in health care workers active in the COVID-19 context. Brazil, 2020 (n=437)

Variable (n)	Univariate						Multiple					
	Critical			Severe			Critical			Severe		
	OR^*^	95% CI^†^	p-value	OR^*^	95% CI^†^	p-value	OR^*^	95% CI^†^	p-value	OR^*^	95% CI^†^	p-value
**Gender**												
Male	0.76	0.49 - 1.19	0.233	1.17	0.54 - 2.56	0.691	0.87	0.52 - 1.46	0.594	1.39	0.58 - 3.30	0.459
Female	1	---	---	1	---	---	1	---	---	1	---	---
**Age group in years old**												
Up to 37	1.14	0.77 - 1.69	0.518	1.37	0.65 - 2.88	0.412	0.97	0.61 - 1.54	0.891	1.33	0.58 - 3.02	0.499
38 or more	1	---	---	1	---	---	1	---	---	1	---	---
**Profession**												
Nursing Tech./Assist.^‡^	1.78	0.89 - 3.55	0.1	2.51	0.85 - 7.40	0.095	1.82	0.82 - 4.07	0.142	3.36	0.99 - 11.39	0.052
Others	1	---	---	1	---	---	1	---	---	1	---	---
**Demand-control**												
High-strain	3.67	2.23 - 6.06	<0.001	7.03	3.18 - 15.67	<0.001	2.55	1.46 - 4.47	0.001	4.57	1.93 - 10.84	<0.001
Others	1	---	---	1	---	---	1	---	---	1	---	---
**Mental distress**												
Yes	4.45	2.84 - 6.97	<0.001	5.1	2.02 - 12.86	<0.001	2.83	1.71 - 4.66	<0.001	2.41	0.88 - 6.58	<0.001
No	1	---	---	1	---	---	1	---	---	1	---	---
**Support at work**												
High	1	---	---	1	---	---	1	---	---	1	---	---
Low	6.25	4.03 - 9.70	<0.001	20.77	6.13 - 70.37	<0.001	4.65	2.91 - 7.44	<0.001	16.82	4.75 - 59.58	<0.001

^*^OR = Odds ratio; ^†^95% CI = 95% Confidence Interval; ^‡^Nursing Tech./Assist. = Nursing Technician or Assistant

## Discussion

When evaluating the distress and pleasure indicators in health care workers on the front line of care, it was identified that most of the professionals under study were in mental distress (61.6%), evidencing that, during evolution of the pandemic in Brazil, distress presents critical levels. This scenario indicates the potential for low pleasure and high distress, in addition to presenting severe professional burnout.

The COVID-19 pandemic has imposed a sudden and impacting change in the work process on health care workers. The increase in the workload in health services, added to the lack of Personal Protective Equipment, the absence of established protocols and the fear of contamination, produced mental distress in health professionals who work on the front line^13^.

In this context, the mental distress perceived by the workers was identified in scientific productions carried out worldwide^13^ ^-^ ^15^. In Brazil, a study that compared psychological symptoms among the categories of health care workers observed a high prevalence of depression, anxiety and stress symptoms and a psychological impact in all categories^16^.

Mental illness in health care workers is not unprecedented, as it has been target of scholars from different countries, covering multiple professionals such as nurses and doctors, as well as medical students, among others. The very nature of the action object of these professionals, exemplified by the constant proximity to suffering, pain and death, has been one of the major causes for distress.

However, since the last two decades, the World Health Organization has signaled its concern with human resources in health, prioritizing aspects such as training, retention, remuneration, appreciation and improvement of the working conditions in its agendas, with goals even to combat precariousness of the work in health^17^.

Therefore, the COVID-19 pandemic takes place in a scenario that already revealed significant concern for the health care workers. Added to this, in a context of constant changes and unpredictability, the risk for increased mental distress is reported in several studies^18^ ^-^ ^19^.

In the current research, the factors that presented a significant association with low to moderate pleasure, in the pandemic context, were high-strain work and the perception of low social support. Allied to these data, among all categories of health care workers active in the front line against the pandemic, nursing technicians and assistants presented a significant association with distress at work. Thus, the results of this study indicate that nursing technicians and assistants experience greater distress at work, in addition to low pleasure due to high-strain work and low social support. 

Corroborating the findings, another recent study, which used the EIPST scale, details that identification with the tasks, freedom to speak at work and solidarity among colleagues are the main revealers for pleasure. On the other hand, stress and exhaustion, as well as feelings of dissatisfaction, injustice, indignation and emotional exhaustion, proved to be the main indicators for distress^20^.

A study carried out with workers in the hemodialysis service related distress to the lack of freedom of expression and recognition at work, with the need for interventions to avoid harms to the workers’ health^21^. Possibly, the social division of work, a characteristic of Nursing, can be an important factor in the genesis of distress in nursing technicians, as social, hierarchy, leadership and power issues are revealed, which can be intensified in critical situations, such as that of the COVID-19 pandemic.

A Brazilian study warns about social inequalities in the pandemic since, in a short period of time, it is observed that the effects of COVID-19 have reached individuals and social groups unequally, even in relation to health care workers. The multiple training and performance path of Nursing professionals in the health services marks the heterogeneous profile of an expressive workforce, often indiscriminate in their technical differences, as different professional categories, such as nursing assistants and technicians, perform equivalent work tasks and often receive remunerations that do not correspond to a distinguished professional education^22^.

In this regard, the current political, economic and social crises in the Brazilian reality, combined with the systematic and conflicting information from the Brazilian government and the media regarding the situation of the evolution of the pandemic and the prevention and treatment measures, in addition to the situation of Brazilian hospitals, increase the feeling of insecurity, uncertainty and lack of control, which can cause mental distress in health care workers^16^. Paradoxically, with the evolution of the pandemic and the intense journalistic coverage of the health care workers’ service, articles were published identifying the professionals as heroes of a war against the SARS-CoV-2 virus. 

In professions considered altruistic, such as those of health care workers, whether medical or nursing professionals, heroism assumes the meaning of the apex of the self-fulfilled professional^23^. Thus, understanding the pandemic, encouragement, a sense of involvement, professional self-fulfillment and the courage to stand firm in the face of confrontation have been observed in health care workers^24^. Considering the scientific literature on the theme, despite the irrefutable importance of the health care workers’ distress, to date, no Brazilian studies have been identified on the pleasure of working in the successive waves of the pandemic. 

A phenomenological study carried out with nurses in China found coexistence of negative and positive emotions. Positive emotions such as confidence, calm and relaxation gradually appeared after the incidence of initial negative emotions. The authors indicate that the positive emotions are possibly related to the nurses’ gradual adaptation, acceptance, positive response and personal growth. They also emphasize that, in order to promote mental health in this pandemic context, it is necessary to strengthen multidimensional social support, to guide positive coping styles, and to stimulate positive emotions^14^.

It is noteworthy that, in the dimension of pleasure on the front line of the Brazilian pandemic, the results of this study point to high pleasure in professional fulfillment, as well as potential for low pleasure in freedom of expression. A study carried out before the pandemic with workers from Basic Health Units, using the Scale of of Pleasure and Distress Indicators at Work (EIPST), showed that professional fulfillment was also satisfactory; however, the freedom factor and lack of recognition presented a serious risk of illness, which can be indicative of injustice, indignation and devaluation for the work performed^25^. 

Another study that used the EIPST scale, before the pandemic and in the Brazilian South region identified that pleasure at work was linked to professional fulfillment with autonomy, freedom and creativity. In this sense, contrary to distress at work, experiences of pleasure and well-being have a strong relationship with being able to express one’s feelings and exercise one’s creativity^26^. 

In the hospital environment, a study evaluated the association between indicators of distress and pleasure at work using the EIPST scale and it was observed that the pleasure indicator was satisfactory. Another significant association in this study was in relation to absenteeism, in which Nursing professionals who were not absent from the service had higher means of pleasure, while higher means of distress were found in those who were absent^27^.

In addition, it is important to highlight the hardship and excess work inherent to the complexity of caring for patients with COVID-19, which makes the service demand higher. It is difficult to intervene in this issue, as the nature of the pandemic imposes this scenario. However, the findings of this study reveal that, despite the difficulties faced in working on the COVID-19 front line, health care workers still feel pleasure in their professional performance, especially due to professional fulfillment. On the other hand, there are aspects that generate distress or reduced pleasure at work that can be modified through management, such as lack of freedom, low social support and lack of recognition. 

The results advance with diverse evidences of the importance of striking a balance between pleasure and distress at work, highlighting the role of management and social support, as lack of support, added to work overload and poor performance management, generates experiences of distress^28^.

As a limitation of this research, it is highlighted that, despite the national scope of the sample, there is a mismatch between the demographic distribution of the country and the participants’ place of residence, as this is a snowball study with participation of workers with greater access to the Internet.

## Conclusion

This study aimed at evaluating distress and pleasure indicators in health workers on the front line of care for suspected or confirmed COVID-19 cases. The findings indicate critical levels of distress at work, presenting the following as important indicators: severe exhaustion with the work demand triggered by the pandemic, lack of recognition and of freedom, and perception of low social support. It is also worth noting that, in relation to pleasure at work, the professional fulfillment dimensions are at a satisfactory level; however, freedom of expression is critical. 

It is considered that, with progression of the pandemic in Brazil and the increase in the number of deaths among health care workers, the scenario still needs research studies that contemplate the pandemic and post-pandemic period, in order to monitor the advance of distress in workers and the impact on the quality of the care provided to the patients.
